# Floor Vibration Experiment and Serviceability Test of iFLASH System

**DOI:** 10.3390/ma13245760

**Published:** 2020-12-17

**Authors:** Jong Ho Lee, Min Jae Park, Sung Won Yoon

**Affiliations:** 1 Department of Architecture, Seoul National University of Science & Technology, 232, Gongneung-ro, Nowon-gu, Seoul 01811, Korea; structure@seoultech.ac.kr; 2 Department of Civil, Environmental and Architectural Engineering, Korea University, Seoul 02841, Korea; alswo8739@korea.ac.kr

**Keywords:** iFLASH, natural frequency, damping ratio, vibration characteristics, serviceability

## Abstract

Studies on novel composite structures that can decrease floor height and improve constructional efficiency in order to increase spatial efficiency and lease revenue have been actively conducted. An innovative fire-proof, lightweight, absorbed, shallow, and hybrid (iFLASH) system was developed to solve construction site issues, such as improving constructability, reducing construction time, and attaining structural efficiency by reducing the weight of the building structure. This system can shorten the construction duration and decrease the floor height and structural weight, owing to features such as a low thickness and light weight. However, studies on the vibration characteristics of this new floor system have not been performed yet. As the general thickness of the iFLASH system ranges from 25 to 30 mm, it must have a sufficient floor vibration performance in order to be utilized. To evaluate the floor vibration performance of the iFLASH system, an experiment was performed in two buildings where the system was applied. This paper presents the results of the dynamic characteristics and serviceability testing as basic data for the vibration characteristics of the iFLASH system.

## 1. Introduction

High-rise buildings are increasingly defining the architecture of modern society due to an increase in the urban population and facility intensification. Accordingly, studies on novel composite structures have been actively conducted for floor-height reduction and to improve constructional efficiency, thereby increasing spatial efficiency and lease revenue [1,2,3].

The iTECH composite beam [4], TSC composite beam [5], MHS composite beam, and Smart beam [6] in Korea, and a deep deck with an asymmetric beam, Flex Fram [7], and Slim Floor [8] with Half PC were developed to decrease the floor height and improve serviceability in the United States and Europe. However, the system possessed a constructability issue, i.e., the beams had to be embedded inside the slab, thus complicating the structure. Precast concrete (PC) construction resolved this problem by shortening the construction duration and improving the constructional efficiency by decreasing the production of molding compositions and the amount of cast-in-place concrete.

However, some limitations remain, such as the heavy weight, the concrete-topping placement, and the need for skilled workers to install joint connections. In particular, the PC method possesses a drawback of low economic feasibility, in contrast to the cast-in-place method, owing to the additional costs resulting from the extra processes and freight charges.

**Figure 1:**
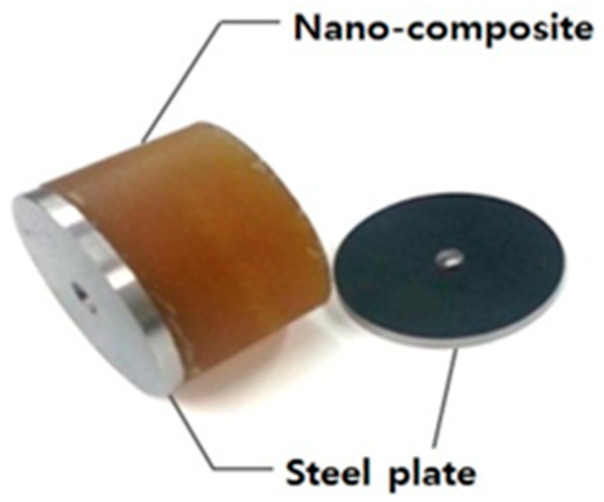
Components of iFLASH system.

Therefore, this study investigated the vibration characteristics of a novel floor system that attains structural efficiency by improving constructability, shortening the construction duration, and reducing the self-weight. The novel floor system investigated is an innovative, fire-proof, lightweight, absorbed, shallow, and hybrid system, i.e., the iFLASH system [9], consisting of a nano-composite between the upper and lower steel plates, as shown in Figure 1.

The nano-composite, with a high bond strength performance, allows this system to exhibit a high bending resistance and reduced self-weight. This is possible because it has a lower density than conventional structural materials. This nano-composite is a type of polymer classified as a polyurethane that is produced with a diisocyanate–polyol mixture. The density of this new material is 1178 kg/m^3^; other mechanical properties of this material are listed in Table 1. Owing to the large rupture strain and high bond strength, this nano-composite can be used in structural elements with steel plates as the iFLASH system.

The system used both bolted joints as well as welding for installation on buildings, and this decreased the construction duration, floor height, and structural weight owing to the low-thickness and lightweight features [10].

Bond strength was a significant factor for securing the structural performance of the module, and it facilitates the demonstration of a fully composite behavior for the iFLASH system even under high deformation [10].

**Figure 2:**
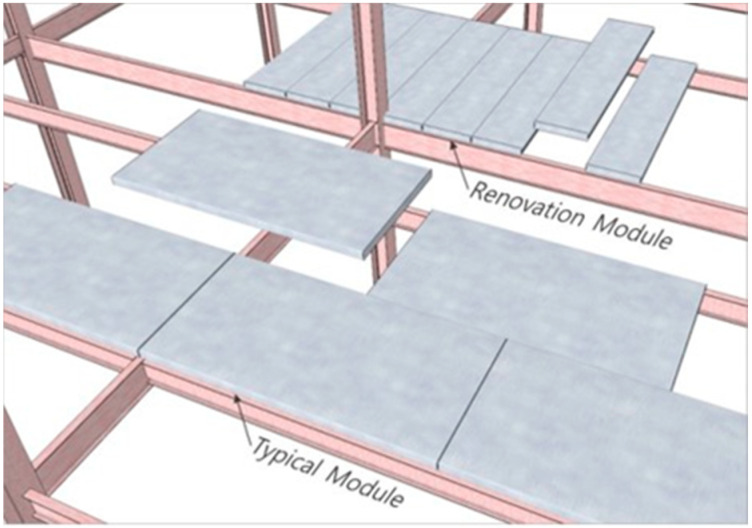
Typical and renovation module.

The sizes of a typical module and a renovation module of the iFLASH system are shown in Figure 2. A typical large module with an approximate area of 15 m^2^ was appropriate for a new building. A small renovation module with an approximate area of 1.5 m^2^ was appropriate for a human to lift [10].

This study mainly investigated the vibration characteristics of the renovation module of the iFLASH system. The international criteria for the serviceability evaluation of floor vibration include housing performance [11,12,13,14]. 

In the criteria specified in [11,12,13,14], the floor’s damping ratio was considered, and high values for vibration criteria were allowed as the damping ratio was increased for floor vibration evaluation. In the American Institute of Steel Construction (AISC) design guide [15], the damping ratio of the steel frame floor slab was suggested to be between 2 and 5% [16].

However, the steel house with a 1 mm (±) surface treatment using light gauge steel that was widely used in the USA and Australia had many partition walls connected to the ceiling height; a screw was used rather than welding, and a high-strength bolt, different from that used in the office steel-frame building, was also used. For this reason, the damping ratio of the walking vibration for the steel house was 9.14%. This value is higher than the 2–5% damping ratio of the steel-frame office buildings. As shown above, the damping ratio varied significantly according to the steel-frame system [16].

Recently, there have been attempts to use the iFLASH system instead of the deck plate slab of an existing steel frame. It is known that this system has many advantages, such as the constructability being comparable to that of deck plate slabs, the lower construction expenses due to the 10% decreased structural weight and 25% reduced construction duration, and economic efficiency [17].

In terms of flexural performance, the iFLASH system exhibits fully composite behavior under large deformations [18], because of the high strength of the bond between the nano-composite and steel plates and the high ductility of the nano-composite. Thus, the upper and lower steel plates in the cross-section mostly resist the bending stress, and the nano-composite perfectly transfers the bending stress from the upper steel plate to the lower steel plate. Therefore, an iFLASH system has an extremely lower thickness than existing slabs, such as steel deck concrete slabs. In addition, several studies have investigated the fire resistance characteristics of the iFLASH system by adding some additives to the nano-composite [19,20,21].

Although the damping ratio of the member unit for this new system was 1.26% [17], the damping ratio of the system installed on the actual buildings was not investigated. The general thickness of the iFLASH system was 25–30 mm, and it was necessary to have sufficient floor vibration performance in order to apply it as a floor system.

Because the thickness of the system was very low compared with that of existing slabs, the floor-vibration performance was a primary concern. Consequently, member-unit floor vibration tests were conducted in [17], and it was concluded that the iFLASH system can be applied to buildings with sufficient floor-vibration performance.

In this study, the iFLASH system was applied to actual buildings, and the floor-vibration performance of the system in actual buildings was investigated via vibration tests. Based on the measured damping ratios, natural frequency, and peak accelerations, the floor-vibration performance of the system was evaluated based on the criteria of the International Organization for Standardization (ISO) and the Architectural Institute of Japan (AIJ) criteria.

## 2. Theoretical Considerations of Vibration

### 2.1. Damping Ratio

In this study, the logarithmic decrement method was applied to calculate the damping ratio of the system [22]. The logarithmic decrement is a formula that shows the damping ratio of the amplitude of an object; the damping ratio of the amplitude at the point where an *n* number of cycles had passed was determined by taking the natural logarithm as shown in Formula (1) [17].

(1) $$\xi =\frac{\ln\left(A/B\right)}{2_{n\pi }}$$

**Figure 3:**
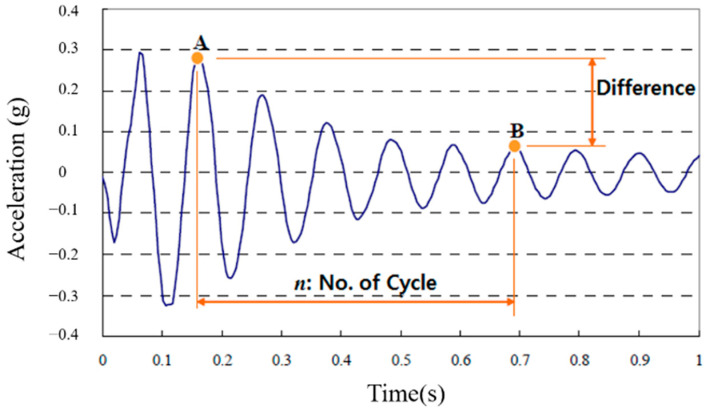
Logarithmic decrement.

In the formula, *A* and *B* are the amplitudes and *n* is the number of cycles. Figure 3 shows the logarithmic decrement. We used the first mode of the free-vibration time-series waveform in order to estimate the damping ratio [23]. 

### 2.2. Vibration Criteria

**Figure 4:**
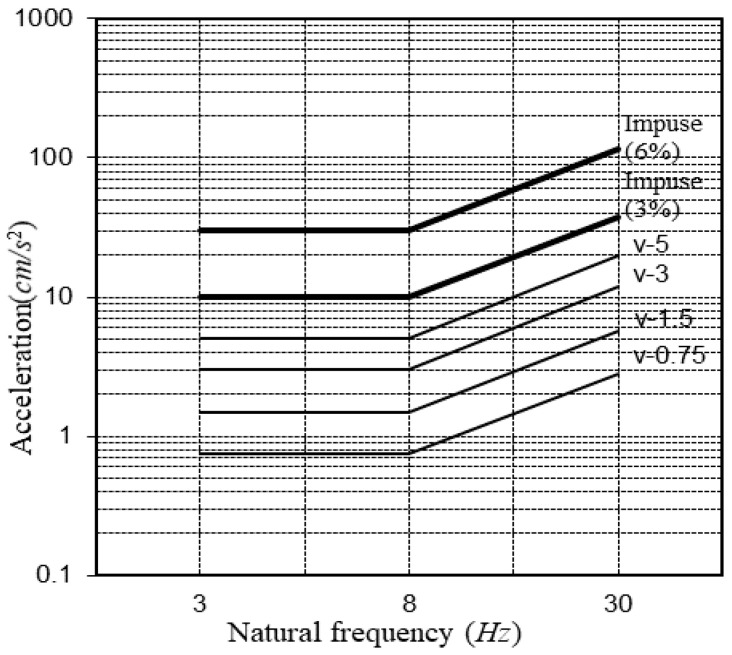
Japanese guidelines [11].

The damping ratio of the floor during the vibration evaluation was considered for the evaluation of habitability based on the building vibration guidelines AIJ, GSA, CSA, and Modified Meister. The vibration criteria can be explained using the AIJ graph shown in Figure 4, and it shows that when the damping ratio of the impulse load increases from 3 to 6%, the allowable acceleration triples within the vibration range from 3 to 30 Hz [11]. Moreover, the CSA criteria [13] (1989) suggested 3, 6, and 12% damping ratios in a similar way that allowed larger criteria vibration values for the walking vibration than continuous vibration on the vibration evaluation curve.

## 3. Experiment Plan and Method

### 3.1. Experiment Plan

The impulse load and walking load were selected as the sources of vibration for two different experiments. The natural frequency was analyzed for the walking load, and the damping ratio was analyzed for the heel impulse (impulse load) [24].

In order to analyze the vibration characteristics according to the construction stage (frame completion and cladding completion), this study investigated the effect of the bond of members and finishing materials on the natural frequency and damping ratio at the various construction phases of actual buildings [24].

### 3.2. Experiment Method

**Figure 5:**
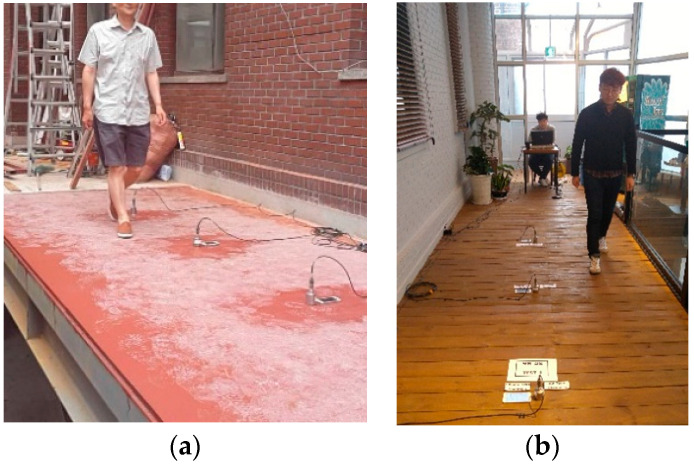
Vibration tests (walking loads): (**a**) Frame completion; (**b**) Cladding completion.

A sensor for measurement was installed at the center of the floor because the response of the floor was the largest at this location [22]. The frame completion is shown in Figure 5a, and the cladding completion is shown in Figure 5b.

In order to investigate the vibration characteristics according to the construction stage, a person weighing 70 kg walked on the floor, 30 cm in the horizontal direction from the sensor, while maintaining a vibration of 2 Hz [25] using a metronome. A heel impulse load was used to measure the damping ratio. The specifications of the equipment [17] used in this study are listed in Table 2.

## 4. Floor Vibration Experiment

### 4.1. Nambuk Church

#### 4.1.1. Natural Frequency

The power spectra of the floor after frame construction completion with the iFLASH system are presented in Table 3. The final values presented here are averages of the values obtained by performing the experiments thrice [23].

The average value of the natural frequency after frame construction completion was 27.6 Hz, and the average value of the natural frequency after cladding completion was 28.5 Hz. There was a difference of 3.26% between the two values.

The natural frequencies according to the construction stage after frame completion and cladding completion are presented in Table 4.

**Figure 6:**
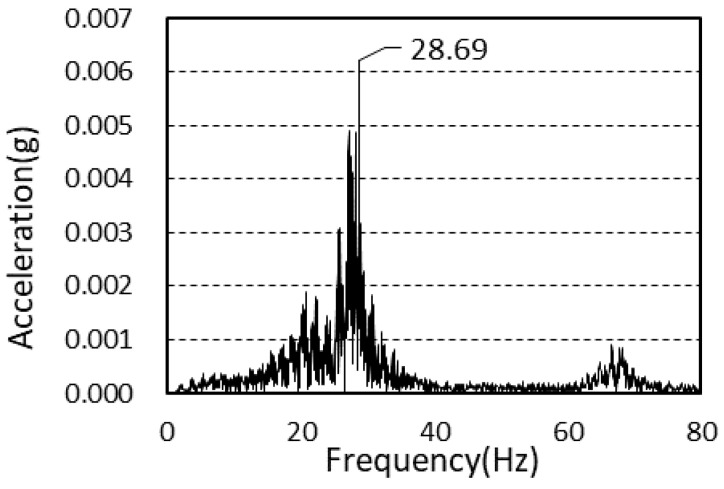
Power spectrum (framing completion).

**Figure 7:**
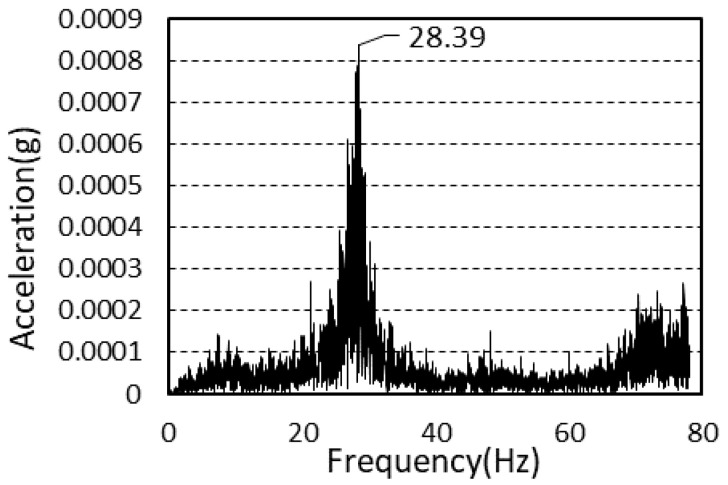
Power spectrum (cladding completion).

Figure 6 and Figure 7 shows the power spectra after framing completion and cladding completion. 

#### 4.1.2. Damping Ratio

**Figure 8:**
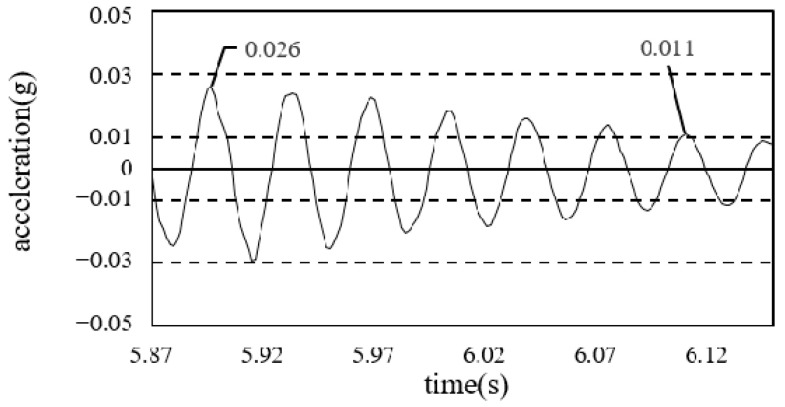
Damping ratio (framing completion).

**Figure 9:**
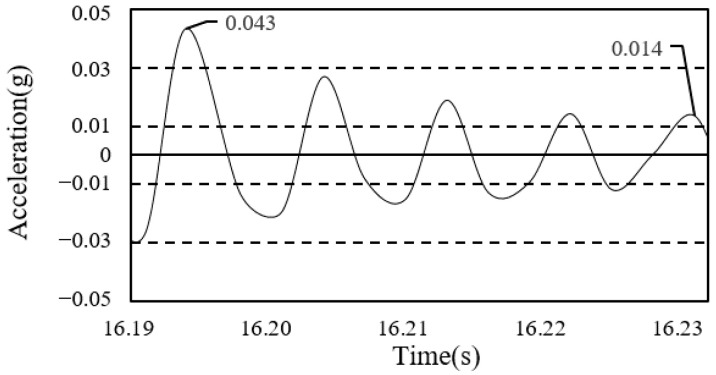
Damping ratio (cladding completion).

To calculate the damping ratio of the floor, the first mode of the free-vibration time-series waveform was used, as shown in Figure 8 and Figure 9.

The values were obtained by performing the experiments thrice. The damping ratios are presented in Table 5.

Considering that the damping ratio of the steel frame floor (which generally utilized the existing deck plate) was between 2 and 5%, it was determined that the iFLASH system had the same damping performance as the existing steel frame.

For the single and assembled members, the damping ratios were 1.26 and 1.17%, respectively [18], and the measured damping ratio after frame completion was 2.63%. After the completion of construction, the damping ratio was 4.57%.

### 4.2. Sangsoo-Dong Building

#### 4.2.1. Natural Frequency

The experiment for the floor vibration was conducted after frame completion and cladding completion on the second and third floors. All experiments were performed thrice and evaluated using the average values. 

For the power spectrum of the slab for the different construction stages, the final values were obtained from three walking vibration values, as presented in Table 6.

The natural frequencies of the vibration of the second and third floors after framing completion were 21.29 and 24.29 Hz, respectively. The average natural frequencies of the vibration of the second and third floors after cladding completion were 21.23 and 20.01 Hz, respectively. 

**Figure 10:**
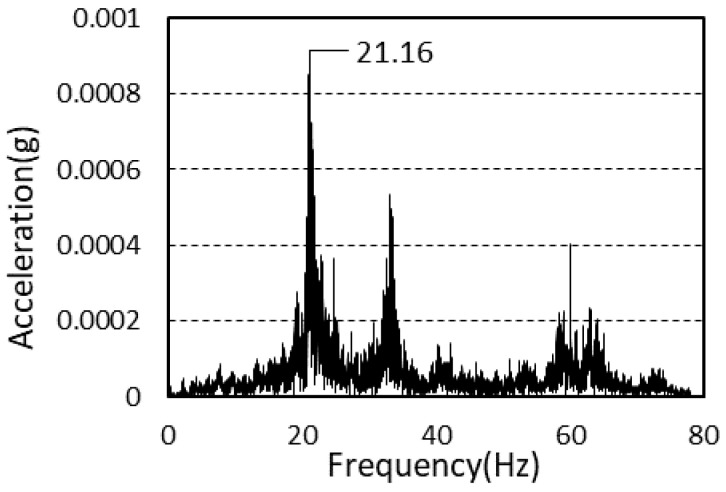
Power spectrum (framing completion).

**Figure 11:**
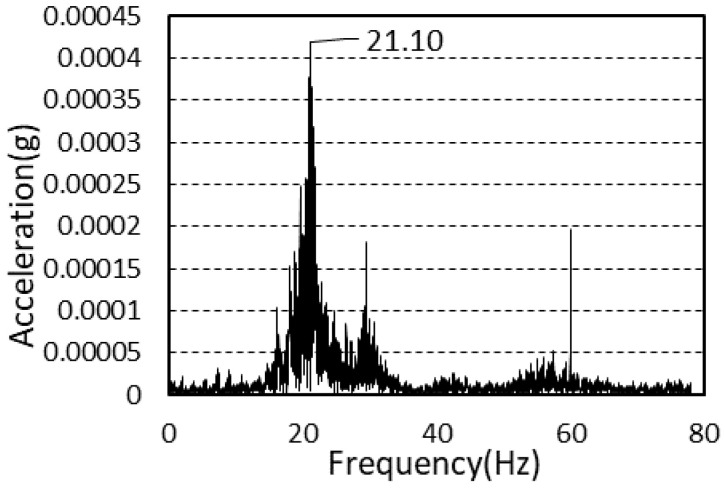
Power spectrum (cladding completion).

The power spectra after framing completion and cladding completion are presented in Figure 10 and Figure 11.

#### 4.2.2. Damping Ratio

**Figure 12:**
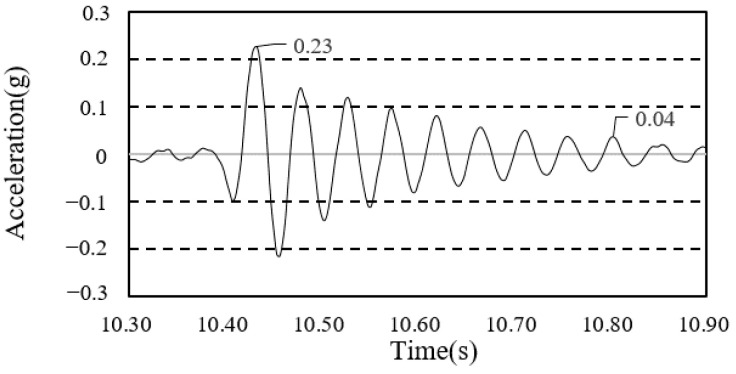
Damping ratio (framing completion).

**Figure 13:**
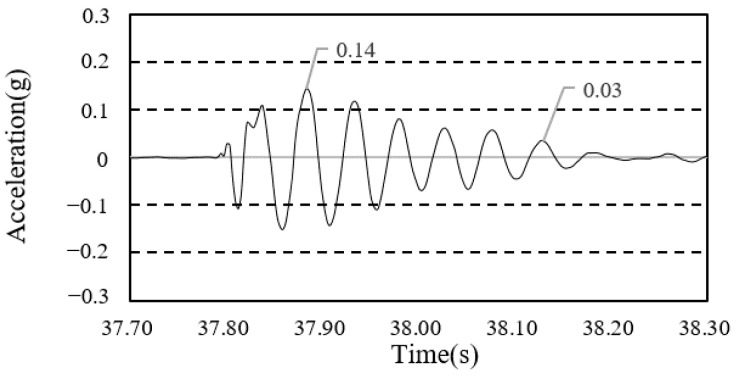
Damping ratio (cladding completion).

The heel impulse test was conducted in order to obtain a free-vibration damping ratio curve. In order to calculate the damping ratio of each construction phase, the first mode of the free-vibration time-series waveform was used, as shown in Figure 12 and Figure 13. The average values obtained by performing each experiment thrice are presented in Table 7.

The measured damping ratio after framing completion was 3.98%, and that after the completion of cladding was 4.90%. The values confirmed that the Sangsoo-dong site had the same damping performance as the existing deck plate.

## 5. Serviceability Test

### 5.1. Maximum Acceleration

To evaluate the maximum acceleration of the iFLASH system, the acceleration of the floor vibration was measured upon the application of a walking load to it. The experiment was conducted thrice after framing and cladding completion on the second and third floors, and the average values of the maximum acceleration are presented in Table 8 and Table 9.

The average maximum acceleration after framing completion for the Sangsoo-dong building was 0.0163 g on the second floor and 0.0170 g on the third floor. The average maximum acceleration after cladding completion was 0.0089 g on the second floor and 0.0089 g on the third floor.

The average maximum acceleration after framing completion for the South-North church was 0.075 g, and the average maximum acceleration of the slab after cladding completion was 0.030 g.

Comparing the measured maximum acceleration results after framing completion and the completion of the construction, the values decreased by approximately 40–60% due to the influence of cladding.

It has been illustrated that non-structural elements can have significant effects on the dynamic responses of the floor systems of buildings; evidence for such effects was observed in determining the dynamic parameters of both slender floor systems [26,27] and traditional concrete floor systems, as detailed [28].

**Figure 14:**
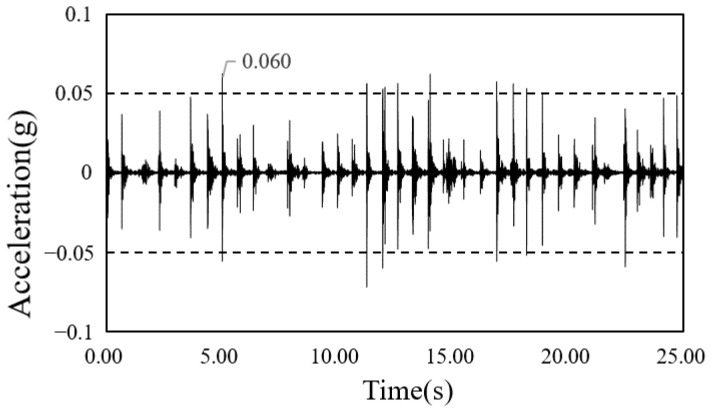
Time history of acceleration after framing completion (South-North church).

**Figure 15:**
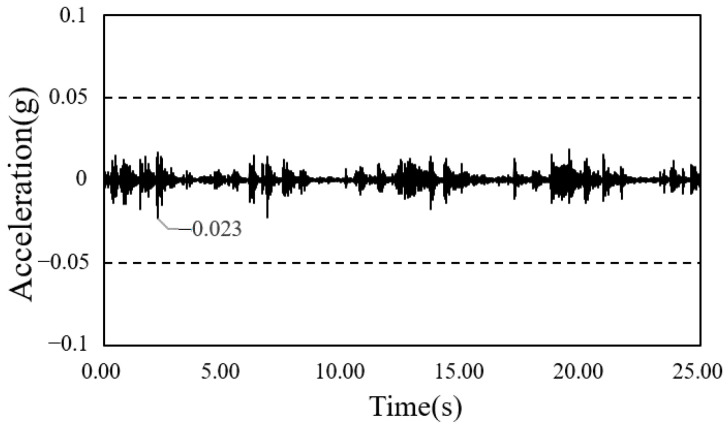
Time history of acceleration after cladding completion (South-North church).

**Figure 16:**
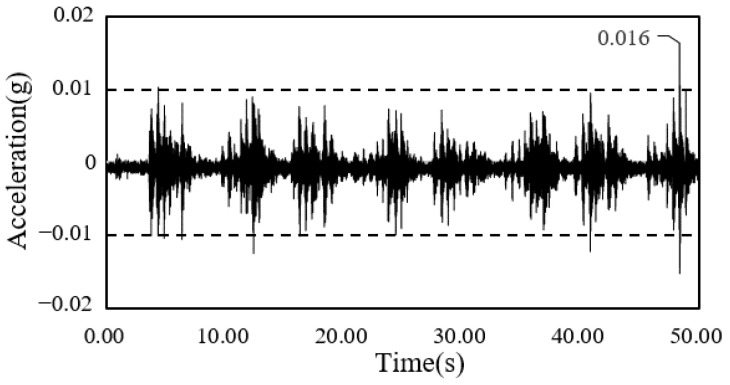
Time history of acceleration after framing completion (Sangsoo-dong building).

**Figure 17:**
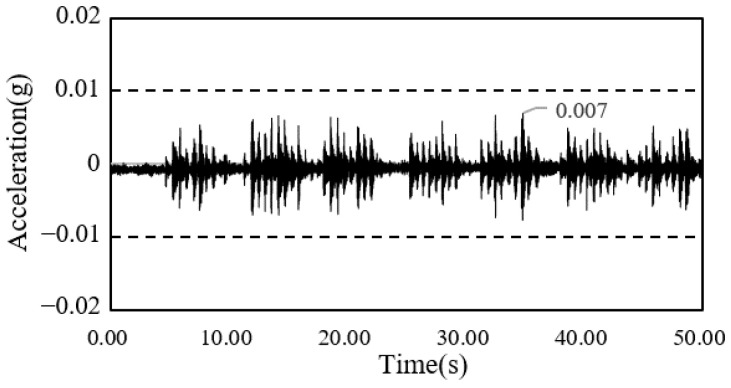
Time history of acceleration after cladding completion (Sangsoo-dong building).

The time-series waveform graphs corresponding to framing completion and the completion of the construction of the South-North church are shown in Figure 14 and Figure 15, and those of the Sangsoo-dong building are shown in Figure 16 and Figure 17.

The vibration evaluation of the iFLASH system was conducted using the guidelines for the evaluation of habitability regarding building vibration [11] and the ISO 2631-211 vibration evaluation standard [29].

### 5.2. Guidelines for the Evaluation of Habitability

The guidelines for the evaluation of habitability [11] for the three categories of vibration are shown in Table 10. The classification of the grades of the allowable vibration range (from V-0.75 to V-30) according to the purpose of the building is shown in Table 11. The walking load was evaluated based on the vibration type 1 (floor affected by continuous or intermittent vibration).

**Figure 18:**
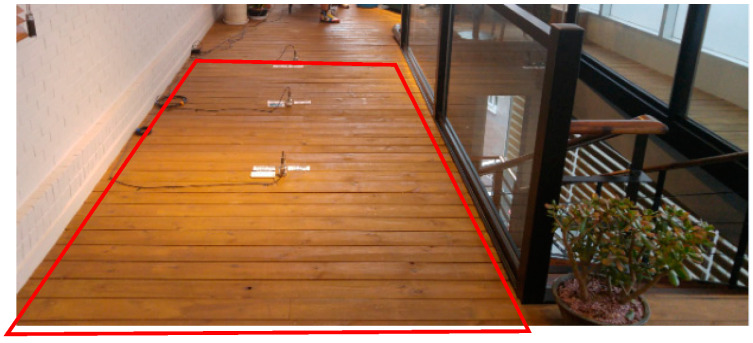
Cladding completion in Nambuk Church.

**Figure 19:**
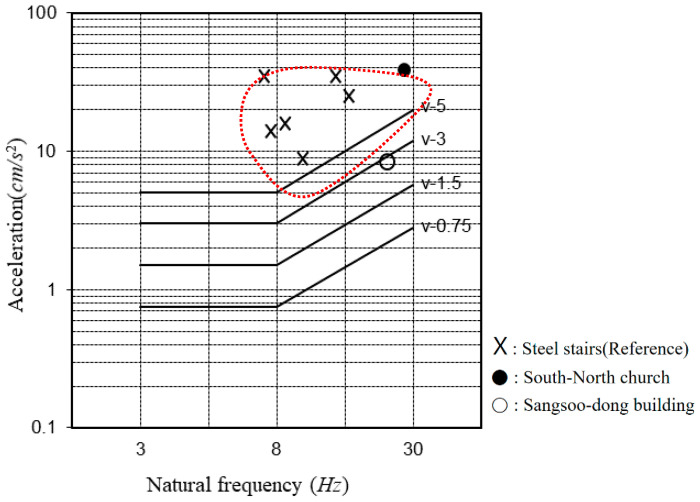
Vibration evaluation curve based on the Architecture Institute of Japan standard [11].

The maximum acceleration in the case of the South-North church was higher than that of the Sangsoo-dong building, which used the same iFLASH system because the three sides of the South-North church were fixed and one side was free due to the installation of stairs (Figure 18). As there was no instruction or criteria for evaluating the vibration of the stairs, the vibrational distribution of the steel frame stairs based on the AIJ vibration criteria was applied (Figure 19).

**Figure 20:**
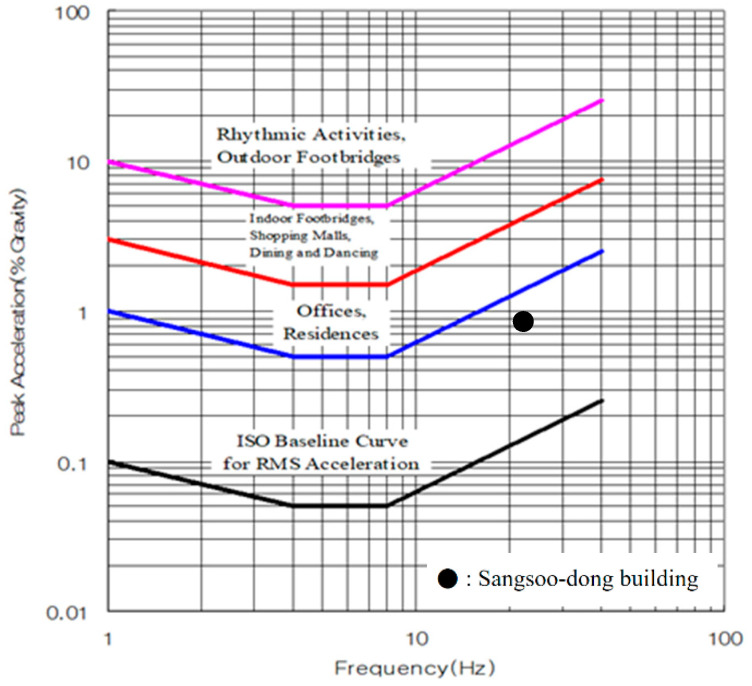
Vibration evaluation curve based on the ISO 2631-2 floor vibration standard [29].

Figure 20 shows the vibration evaluation curve (based on [11]); the floor vibration is evaluated through the natural frequency and the maximum acceleration of the vibration of the floor. The natural frequency and the maximum acceleration of the iFLASH system were determined based on the experimental results for the two buildings, and the values are presented in Table 12.

For the South-North church, the maximum vibration distribution of the X-marked steel frame stair was similar to the maximum acceleration of the slab of the South-North church.

The result of the vibration evaluation for the Sangsoo-dong building is presented in Figure 20, and it satisfies the V-3 vibration performance standard. Therefore, this system met the Grade II standard with respect to housing and the Grade I standard with respect to offices.

### 5.3. ISO 2631-2

ISO 2631-2 suggests a floor vibration standard based on the vibration evaluation curve. Similar to the AIJ [11] standard, it evaluates the vibration performance of a building by the natural frequency and maximum acceleration. The natural frequency and maximum acceleration are presented in Table 13.

The result of the vibration evaluation for the Sangsoo-dong building is presented in Figure 20, and it satisfied the Offices & Residences part of the vibration performance standard.

## 6. Conclusions

In order to investigate the floor vibration characteristics of an iFLASH system, this study performed a floor vibration experiment for walking loads and heel impulses. However, human excitation of the load was used so that the free vibration could be different for each experiment. Thus, according to the serviceability guidelines, one person excited a load as equal as possible. The target buildings where the iFLASH system was applied were the construction site of the South-North church and the new construction site of the Sangsoo-dong building. The iFLASH system was determined to be a new floor system that can effectively play the role of a slab with extremely low thickness and improving constructability, shortening the construction duration, and reducing the self-weight. However, the drawback of the iFLASH system is that it is not commercialized and mass produced at present, so the cost of the materials is high, but the overall construction cost is similar because the installation is simple and it shortens the construction duration. If commercialization and mass production are possible in the future, it is believed that cheaper construction costs will be realized. The system used both bolted joints as well as welding for installation on buildings, and this decreased the construction duration, floor height, and structural weight, owing to the low-thickness and lightweight features. Accordingly, owing to the very low thickness of the system, the floor vibration performance was a point of major concern.

Therefore, floor vibration tests for the iFLASH system in real buildings were conducted to evaluate the vibration performance. However, in this study, the vibration tests were conducted only in commercial facilities; therefore, it is highly recommended for a future study to conduct a vibration test and serviceability test under the same conditions of the iFLASH slab installation for different facilities such as offices, residences, etc. The results are summarized below.

(1) According to the results of the floor vibration experiment at the South-North church construction site, the natural frequency after the completion of the construction was 28.5 Hz, and the damping ratio was 4.57%. For the Sangsoo-dong building, the natural frequency after the completion of the construction was 21.2 Hz, and the damping ratio was 4.90%. Based on the result for a steel frame building with a normal deck plate showing a 2–5% damping ratio, it was found that the iFLASH system had the same damping performance as the existing steel frame.

(2) According to the serviceability test results, the natural frequency was 28.5 Hz, and the maximum acceleration was 0.030 g. As the three sides of the South-North church were fixed and only one side was free because of the stairs installed, its maximum acceleration was greater than that of the Sangsoo-dong building, for the same iFLASH system. As there was no instruction or criteria for the evaluation of the vibration of stairs, the vibrational distribution of the steel frame stairs corresponding to the AIJ vibration criteria was applied with the AIJ guidelines for the evaluation of habitability, and it showed a similar distribution.

(3) For the Sangsoo-dong building, the natural frequency after the completion of construction was 21.2 Hz, and the maximum acceleration was 0.009 g. As this result satisfied the vibration performance criteria of ISO 2631-2 for both offices and residences, the iFLASH system applied in the Sangsoo-dong building can be safely used for current purposes. 

## Figures and Tables

**Table 1:** Mechanical properties of the nano-composite used in this study.

Property	Value
Density	1178 kg/m^3^
Tensile strength	31.4 MPa
Young’s Modulus in tension	1277 MPa
Rupture strain in tension	0.295
Compressive strength	23.1 MPa
Young’s Modulus in compression	461 MPa
Poisson’s ratio	0.39
Bond strength with steel plates	6.34 MPa

**Table 2:** Measurement equipment.

Equipment	Parameter	Specification
ICPAccelerometer(Dytran 3191A, Dytran, Los Angeles, CA, USA)	Capacity	±1 g
	Output voltage	±5 g
	Volt sensitivity	5000 mV/g
	Transversesensitivity	4.2%
Dynamic	Channel	4
Analyzer	Filter	90 dB
(Signalcalc Mobilyzer)	Acquisition	Approximately 5–8 Hz
Metronome(SQ 10–77, Seiko, Tokyo, Japan)	Approximately 30–250 times/min	Approximately 30–250 times/min

**Table 3:** Natural frequency.

Construction Phase	Natural Frequency (Hz); Experimental Value, *E*
Framing completion	27.6
Cladding completion	28.5

**Table 4:** Natural frequencies for different construction phases.

Construction Phase	Natural Frequency (Hz)			
	1st	2nd	3rd	Average
Framing completion	28.69	27.18	26.86	27.6
Cladding completion	28.39	28.63	28.41	28.5

**Table 5:** Damping ratios of specimens [19].

Construction Phase	Damping Ratio (%)
iFLASH panel	1.26
iFLASH composite panel	1.17
Framing completion	2.63
Cladding completion	4.57

**Table 6:** Natural frequency of the floors for different construction phases.

Construction Phase		Natural Frequency (Hz)			
		1st	2nd	3rd	Average
Frame completion	2nd floor	21.16	21.30	21.40	21.29
	3rd floor	24.72	24.78	23.37	24.29
Cladding completion	2nd floor	21.10	21.30	21.30	21.23
	3rd floor	21.24	19.11	19.69	20.01

**Table 7:** Damping ratio of specimens.

Specimens	Damping Ratio (%)
Frame completion	3.98
Cladding completion	4.90

**Table 8:** Maximum acceleration (Sangsoo-dong building).

Construction Phase		Max Acceleration (g)			
		1st	2nd	3rd	Avg.
Frame completion	2nd floor	0.0161	0.0153	0.0175	0.0163
	3rd floor	0.0173	0.0169	0.0167	0.0170
Cladding completion	2nd floor	0.0088	0.0095	0.0084	0.0089
	3rd floor	0.0070	0.0098	0.0099	0.0089

**Table 9:** Maximum acceleration (South-North church).

Construction Phase	Max Acceleration (g)			
	1st	2nd	3rd	Avg.
Frame completion	0.060	0.079	0.087	0.075
Cladding completion	0.023	0.029	0.038	0.030

**Table 10:** Japanese guidelines [11].

Vibration Type	Details
1	Floor affected by continuous or intermittent vibration
2	Floor with low damping characteristics affected by impact vibration, damping ratio under 3%
3	Floor with high damping characteristics affected by impact vibration, damping ratio between 3 and 6%

**Table 11:** Natural frequency and maximum acceleration [11].

Grade According to Building Type		Vibration Type 1			Vibration Type 2	Vibration Type 3
Building Practicability		Grade I	Grade II	Grade III	Grade III	Grade III
Housing	Dining, bedroom	V-0.75	V-1.5	V-3	V-5	V-10
Office	Meeting room	V-1.5	V-3	V-5	V-10	V-30
	General office	V-3	V-5	Approximately V-5	Approximately V-10	Approximately V-30

**Table 12:** Natural frequency and maximum acceleration for the two buildings.

Buildings	Natural Frequency (Hz)	Max. Acceleration (cm/s^2^)
Nambuk Church	28.5	30
Sangsoo-dong	21.2	8.9

**Table 13:** Natural frequency and maximum acceleration.

Natural Frequency (Hz)	Maximum Acceleration (%g)
21.2	0.9
